# Swedish translation and psychometric testing of the safety attitudes questionnaire (operating room version)

**DOI:** 10.1186/1472-6963-13-104

**Published:** 2013-03-19

**Authors:** Camilla Göras, Fan Yang Wallentin, Ulrica Nilsson, Anna Ehrenberg

**Affiliations:** 1 Anesthesia and Intensive Care Unit, Falu Lasarett, Falun, Sweden; 2 Department of Statistics, Uppsala University, Box 513, Uppsala, Sweden; 3 School of Health and Medical Sciences, Örebro University, Örebro, Sweden; 4 School of Health and Social Studies, Dalarna University, Falun, Sweden

**Keywords:** Patient safety, Operating room, Safety climate, Psychometrics, Translation, Safety attitudes questionnaire

## Abstract

**Background:**

Tens of millions of patients worldwide suffer from avoidable disabling injuries and death every year. Measuring the safety climate in health care is an important step in improving patient safety. The most commonly used instrument to measure safety climate is the Safety Attitudes Questionnaire (SAQ). The aim of the present study was to establish the validity and reliability of the translated version of the SAQ.

**Methods:**

The SAQ was translated and adapted to the Swedish context. The survey was then carried out with 374 respondents in the operating room (OR) setting. Data was received from three hospitals, a total of 237 responses. Cronbach’s alpha and confirmatory factor analysis (CFA) was used to evaluate the reliability and validity of the instrument.

**Results:**

The Cronbach’s alpha values for each of the factors of the SAQ ranged between 0.59 and 0.83. The CFA and its goodness-of-fit indices (SRMR 0.055, RMSEA 0.043, CFI 0.98) showed good model fit. Intercorrelations between the factors safety climate, teamwork climate, job satisfaction, perceptions of management, and working conditions showed moderate to high correlation with each other. The factor stress recognition had no significant correlation with teamwork climate, perception of management, or job satisfaction.

**Conclusions:**

Therefore, the Swedish translation and psychometric testing of the SAQ (OR version) has good construct validity. However, the reliability analysis suggested that some of the items need further refinement to establish sound internal consistency. As suggested by previous research, the SAQ is potentially a useful tool for evaluating safety climate. However, further psychometric testing is required with larger samples to establish the psychometric properties of the instrument for use in Sweden.

## Background

Globally, tens of millions of patients suffer disabling injuries or death every year due to unsafe medical care [1]. A landmark paper from the Institute of Medicine (IOM) concluded that healthcare in the United States is not as safe as it should and could be. At least 44 000, and probably as many as 98 000, people die in hospitals in the US because of medical errors [2]. The Swedish Ministry of Health and Social Affairs estimates that almost 100 000 patients are affected every year by preventable injuries. Half of all preventable injuries occur in connection with surgery or other invasive procedures [3]. According to IOM and the World Health Organization (WHO), medical errors have an important economic burden as they may result in prolonged hospitalization and loss of income [1,2]. Medical errors may also result in detrimental loss of trust in the healthcare system [2].

Error is defined as “an act of commission (doing something wrong) or omission (failing to do the right thing) that leads to an undesirable outcome or with significant potential for such an outcome” [4]. However, some errors do not result in adverse events, and these are often characterized as “near misses”. A health care injury is often a consequence of an adverse event, which is preventable in most cases [5]. An adverse event is defined as an injury resulting from a medical intervention (i.e., not due to the underlying medical condition) [2]. The basis of all patient safety work is knowledge of the organization’s risks and awareness of the system’s shortcomings [6]. Patient safety is characterized as a nationwide problem and, to improve patient safety, the focus should be on the processes of care instead of blaming individuals [2,6].

The concept of safety culture was launched in connection with the Chernobyl nuclear accident in 1986 [7]. Safety culture exists in all health care organizations, and a high level of patient safety is dependent on whether the organization has a positive safety culture [8]. The definition of safety culture and climate has been debated [9,10]. Safety climate has been described as employees’ perceptions, attitudes, and beliefs about risk and safety. Safety culture is a more complex concept that reflects fundamental values, norms, and expectations [11]. Therefore, the term “climate” is used in this report.

Safety climate surveys are increasingly being used within health care organizations [12]. Twelve tools for measuring patient safety were designated for health care, including the widely used Safety Attitudes Questionnaire (SAQ) and Hospital Survey on Patient Safety (HSOPS) based on their validity, reliability, and the links established between the survey and positive patient outcomes [13]. The SAQ is the only survey tool that demonstrates a link between good survey results and reduced health care-associated infections [13,14], and it is the most thoroughly validated instrument for assessing the safety climate [15].

Several risk factors that can influence clinical practice were described by Vincent’s theoretical framework in 1998, including the organization (i.e. safety climate); work environment (i.e. staffing levels and workload); team-; and individual staff (i.e. overconfidence) [16]. Factors that may constitute a threat to patient safety among registered nurses (RNs) working in the operating room (OR) setting include a perceived imbalance in staffing [17] and increased speed of work [17-19]. Patient safety itself accounts for the greatest stress among RNs working in the OR [20]. The OR environment has been described as a high-risk environment for patients and one of the most complex work places within health care due to its sophisticated technology and the involvement of multidisciplinary team members [7,21]. In addition, surgical procedures are often performed in high-risk situations and under time pressure [7].

In summary, patient safety is a central principle of quality in health care and has high priority on the research agenda in most countries. The OR setting has been described as a complex high-risk environment, and some existing safety problem areas exist within the OR. Perceived imbalance in staffing and increased work pace is factors that may threaten patient safety. Measuring the safety climate in a workplace is an important step in understanding and improving patient safety [2,7]. Previous research has assessed the psychometric properties of the SAQ across countries and in different contexts and settings [22-25]. The internal consistency and Cronbach’s alpha values are acceptable [22-24], and the construct validity measured by CFA generally exhibits satisfactory model fit [22-25]. However, a lack of research exists on safety climate in Swedish OR settings. Therefore, an instrument that measures health care professionals’ attitudes regarding safety climate in the OR would be helpful in understanding and identifying areas that need improvement and for evaluating improvements in interventions. The purpose of the present study was to establish the reliability and validity of the translated version of the SAQ (OR version) by evaluating its psychometric properties.

## Methods

### Safety attitudes questionnaire

The SAQ was developed to measure attitudes regarding safety climate. The SAQ is a refinement of the Intensive Care Unit Management Attitudes Questionnaire (ICUMAQ) [26], which was derived from the Flight Management Attitudes Questionnaire (FMAQ) [27]. The FMAQ was created after most airline accidents were found to be due to personal aspects of the crew. The SAQ contains items from the FMAQ and two conceptual models: Vincent’s framework for analyzing risk and safety in clinical medicine, and Donabedian’s conceptual model for assessing quality [25]. The SAQ was adapted for use in intensive care units [28], ORs [29], general inpatient settings, such as medical wards [25], and ambulatory clinics [30]. Every version of the survey contains most of the same items, but with minor changes [25]. The full version of the SAQ comprises 60 items, whereas the OR version contains 59 items, with 30 belonging to six factors [12]. The factors are teamwork climate, job satisfaction, perceptions of management, safety climate, working conditions, and stress recognition. Examples of factor definitions and items are presented in Table 1. Each item is answered using a five-point Likert scale: from “Disagree Strongly” to “Agree Strongly”, “Neutral” and “Not applicable” based on the respondent’s experiences in the OR department where they work.

**Table 1:** SAQ factor definitions and example items

**Factor definitions**	**Example of items**
Teamwork climate: perceived quality of collaboration between personnel	–Disagreements in the ORs here are resolved appropriately (i.e., what is best for the patient).
	–The physicians and nurses here work together as a well- coordinated team.
Job satisfaction: positivity about the work experience	–I like my job.
	–This hospital is a good place to work.
Perceptions of management: approval of managerial action	–Hospital administration supports my daily efforts.
	–Hospital management is doing a good job.
Safety climate: perceptions of a strong and proactive organizational commitment to safety	–I would feel perfectly safe being treated here as a patient.
	–Personnel frequently disregard rules or guidelines that are established for the OR.
Working conditions: perceived quality of the OR’s work environment and logistical support (staffing, equipment, etc.)	–Our levels of staffing are sufficient to handle the number of patients.
	–Medical equipment in the ORs here is adequate.
Stress recognition: acknowledgement of how performance is influenced by stressors	–I am less effective at work when fatigued.
	–When my workload becomes excessive, my performance is impaired.

### Swedish version of the SAQ

The original version of the SAQ was received from the developer and permission obtained to use it. The translation process followed guidelines from the International Society for Pharmacoeconomics and Outcomes Research (ISPOR) [31]. The first translation was made by two of the researchers (CG, AE), one of whom was a RN specializing in anesthesia (CG), in order to reduce the potential bias of each forward translator. Both researchers spoke Swedish as their native language and were proficient in English. The forward translations were reconciled into a single forward translation by the researchers in order to resolve any discrepancies and seek agreement. In addition, as a part of the translation process, the preliminary version was tested on a sample of OR staff (n = 6) with varying specialties and ages. This process resulted in some minor revisions of the questionnaire. These respondents were not included in the forthcoming survey.

A back-translation was made by a professional translator who spoke English as a native language in order to verify the quality of the translation and that the items had the same meaning and semantic equivalence. In order to compare the items between the original SAQ and the back-translation, the items were rated by one of the researchers on a four-point ordinal scale: 1=highly consistent, 2=quite consistent, 3=somewhat consistent, and 4=not consistent. Twenty-seven of the items were highly consistent, 27 were quite consistent, 5 were somewhat consistent, and none of the items were rated as non-consistent. To detect and deal with any translation ambiguities between the versions and to ensure conceptual equivalence between the English and Swedish versions, the research group reviewed and discussed the two versions, resulting in some revisions. The back-translation was then sent to the developer of the original SAQ, followed by a discussion with the developer about problematic items. Two items were excluded in the translated version of the SAQ: “The staff surgeon/attending surgeon should be formally in charge of the operating room staff during a surgical procedure”, as this item is not coherent with the Swedish OR setting. The item “If the respondent has ever completed this survey before”, was excluded as this was the first pilot testing of the instrument. Thus, the final number of items in the Swedish-OR version was 57.

To assess content validity, a validation review was performed by an expert committee, including relevance and intelligibility, to highlight any items that may be inappropriate at a conceptual or cultural level. A purposeful sample of five experts was used, including one physician, three RNs (two with PhD degrees), and one licensed practical nurse (LPN). To evaluate agreement among the experts, a content validity form was developed. To compute a content validity index (CVI) at the item level (I-CVI), the rating of either 3 or 4 on the four- point scale of relevance and intelligibility was divided by the number of experts [32]. The items were rated on a four-point ordinal scale of relevance and intelligibility as follows: 1=not relevant, 2=somewhat relevant, 3=quite relevant, and 4=highly relevant. The expert committee was also asked to comment on the items that were assigned low ratings.

The research group reviewed the results from the expert committee and identified the items that needed to be revised, i.e. items with I-CVI < 0.78, according to suggestions by Polit and Beck [32]. The experts’ comments also resulted in some modifications to the translated version of the SAQ due to cultural differences: I) instead of referring to the hospital as the organizational unit, reference was made to units or the OR department; II) fatigue was perceived as too severe as an expression and was translated as being tired; III) as an example of emergency situations, acute caesarean section was used instead of emergency resuscitation. Based on comments from the experts and communication with the developer of SAQ, the term “hospital management” was exchanged for “unit management”.

### Instrument

The full version of the Swedish translation of the SAQ (OR version) consists of 57 items, with 30 constituting six factors of safety climate included in the psychometric testing in this study. Seven supplementary questions covering the demographics of the respondents, including age, sex, occupational group, and work experience, were also included.

### Participants and setting

Three hospitals in central Sweden participated in the study: two county hospitals and one university hospital. One hospital (A) consisted of different small OR settings and one anesthesia setting that serves all OR settings. The other two hospitals (B, C) had one central OR setting that serves a variety of surgical clinics. The survey was carried out among registered nurse anesthetists (RNAs), OR nurses, and LPNs working in an OR for at least 6 months and who were on duty during the data collection period. A total of 374 staff members within three OR departments were eligible for participation, and 237 (63%) consented to participate. Hospital A had a response rate of 58%, hospital B 79% and hospital C 56%. Of the respondents, 46% were RNAs, 34% OR nurses, and 17% LPNs (3% missing data). A total of 89% of the respondents were female and the mean age of the respondents was 47 years. The respondents had experience working at the hospital a mean 19.9 years, 15.2 years in peri-operative care.

### Procedure

The data collection period was January to March 2011. Data collection differed somewhat between the included hospitals for practical reasons. In two hospitals (B, C) the information about the study was given during staff meetings and the questionnaire distributed to each employee´s postbox. In one of these hospitals (C), a name and number list was used and reminders sent two times to those who had not returned the questionnaire. In the other hospital (B), anonymous questionnaires were used for consideration of the respondents’ integrity because it was the author’s workplace. Reminders were sent twice to all respondents. One hospital (A) consisted of different OR units and a web-based survey was used for practical reasons. The unit managers were informed via e-mail and the questionnaire distributed in an electronic format together with information about the study, and the managers forwarded it via e-mail to the respondents. Three web-based reminders were administered with the assistance of the managers. The questionnaire took approximately 15 minutes to complete.

### Analysis

The internal dropout rate was 1-5% within 22 of the 30 analyzed items. One item within the factor working conditions had 8% of observations missing. A missing value imputation was made before the CFA to overcome the problem of missing values [33]. For more detailed information see Additional file 1.

Cronbach’s alpha was computed to evaluate internal consistency [32]. Two items were negatively worded and reversed for the statistical analysis. An approach to construct validation, CFA, was used for conclusions about the conceptual and semantic equivalence of a translated questionnaire [32], as well as to create other aspects of psychometric evaluation [34]. CFA postulates certain relationships among the observed and the latent variables assuming a pre-specified pattern for the model parameters, such as factor loadings, factor correlations and error variances. CFA is mainly used for testing hypothesis arising from theory. Therefore, the number of factors (latent variables) and number of observed variables (indicators) that used to measure the latent factors are determined in advance. This implies that the analyst has enough knowledge to formulate the model hypothesis, i.e., the relationships between the latent factors or latent factors and the observed indicators that they explain [35]. The goodness-of-fit statistic was used to measure whether the overall model fit was good [32]. Each fit class (absolute, parsimony, and comparative) in the goodness-of-fit analysis provides different information about the model fit. At least one index from each fit class should be analyzed because each provides different information about the fit of the CFA solution [34]. Three different fit indices were used: standardized root mean square residual (SRMR), root mean square error of approximation (RMSEA), and comparative fit index (CFI). A good model fit between the target model and the observed data is distinguished by SRMR values between 0.0 and 1.0, where 0.0 indicates perfect fit, and RMSEA values ≤ .05 and CFI values ≥ .95 [34]. The goodness-of-fit statistics and correlation matrix were analyzed by the program Linear Structural Relations analysis (LISREL) 8.80 [36]. SPSS version 13.0 was used for evaluating internal consistency.

### Approval of ethics committee

The Directors of the OR departments gave their permission to perform the data collection. The Research Ethics Committee at Dalarna University, Sweden, approved the study. The participants were informed that participation was voluntary, that all answers would be treated with confidentiality, and that their participation would not have any impact on their working conditions.

## Results

### Internal consistency

The internal consistency of the six factors and 30 items of the translated version of the SAQ had Cronbach’s alpha values of 0.59 to 0.83. Safety climate had the highest Cronbach’s alpha values, and working conditions had the lowest values (Table 2).

**Table 2:** Internal consistency for the six factors of SAQ

**SAQ factors**	**Cronbach’s alpha**
Safety Climate (7 items)	.83
Teamwork Climate (6 items)	.80
Job Satisfaction (5 items)	.78
Stress Recognition (4 items)	.76
Perceptions of Management (4 items)	.63
Working Conditions (4 items)	.59

### Internal construct validity

The goodness-of-fit values used to evaluate the internal construct validity are displayed in Table 3. The SRMR value was 0.055, the RMSEA values were below the recommended limit of 0.05, and the CFI value was well above 0.95, which indicates a good model fit approximation of the translated version of the SAQ. The test of the hypothesized relationships among the factors and items showed that the correlation ranged from 0.92 to 0.70 and that five of the six factor correlations were significant. Stress recognition had a negative correlation with all other factors, and no significant correlation was found between stress recognition and teamwork climate, job satisfaction, or perception of management on the 1% level. Safety climate and working conditions significantly correlated with stress recognition on the 5% level. The intercorrelations between the factors are presented in Table 4.

**Table 3:** Goodness-of-fit indices for CFA of the SAQ factors

**Sample size**	**237**
Standardized root mean square residual (SRMR)	0.055
Root mean square error of approximation (RMSEA)	0.043
Comparative fit index (CFI)	0.98

SRMR reference: 0.0 to 1.0, with 0.0 indicating perfect fit. RMSEA reference: ≤ 0.05 = good, ≥ 0.10 = poor fit. CFI reference: 0.90-0.95 = acceptable, > 0.95 = good.

**Table 4:** Correlation matrix for the SAQ factors

	**Safety climate**	**Teamwork climate**	**Job satisfaction**	**Stress recognition**	**Perception of management**	**Working conditions**
Safety Climate	1.00					
Teamwork Climate	0.88 (6.56^***^)	1.00				
Job Satisfaction	0.70 (5.82^***^)	0.89 (6.29^***^)	1.00			
Stress Recognition	-0.23 (-2.60^**^)	-0.08 (-0.93)	-0.03 (-0.36)	1.00		
Perceptions of Management	0.78 (6.13^***^)	0.87 (6.39^***^)	0.83 (6.09^***^)	-0.16 (-1.70)	1.00	
Working Conditions	0.85 (6.27^***^)	0.91 (6.40^***^)	0.89 (6.14^***^)	-0.23 (2.25^**^)	0.92 (6.32^***^)	1.00

Values in parentheses are T-values.

** Significant on 5% level.

*** Significant on 1% level.

### SAQ factors and item descriptives

The SAQ factor definitions and items; missings, mean, median, agree (agree strongly) and disagree (disagree strongly) answers is described in Additional file 1.

## Discussion

The present study represents the first report of the attitudes regarding safety climate in ORs in Sweden. The internal consistency and internal structure of the Swedish translation of the SAQ (OR version) was assessed; the translation showed satisfactory psychometric properties with comparable benchmarking data [25]. Similar research has shown that these results are comparable with somatic clinical areas in a Norwegian hospital and pharmacies in Sweden [22,23]. However, the reliability analysis suggested that some items in the Swedish version need further refinement to establish sound internal consistency. The construct validity, which is judged by the CFA using goodness-of-fit indices, showed good model fit. The correlation between all factors showed moderate to high correlation except for stress recognition, which had no significant correlation with teamwork climate, perceptions of management, or job satisfaction. This pattern was also found in previous studies [23,25].

### Internal consistency

The internal consistency for the six factors of the Swedish version of the SAQ in terms of Cronbach’s alpha values ranged from 0.76 to 0.83, except for the factors perception of management and working conditions. Perception of management had an alpha value of 0.63, which is a bit below the recommended acceptable alpha value limit of 0.70 [37]. Notably, the items within the factor perception of management had several missing data, which might have impacted the alpha value. Working conditions scored 0.59, which is below the acceptable limit. This Cronbach’s alpha value might indicate that some items within this factor have to be revised. The findings from this study should be taken into consideration in further evaluations of the reliability of the SAQ for use in Sweden. Cultural aspects may exist regarding the perception of management though changes were made to accommodate the Swedish setting.

The Cronbach’s alpha values for teamwork climate and safety climate were relatively high compared to a previous study of Swedish pharmacies [23] in which perception of management and working conditions represented the lowest alpha values, in concordance with the present study. However, the values were above the acceptable limit of 0.70. The second lowest alpha values in the Norwegian study, which represents a similar context as Sweden, were found for working conditions, but the Norwegian Cronbach’s alpha values were just above the acceptable limit. The factor teamwork climate scored below the limit in the previous study [22], but achieved high values in this study. This difference may reflect that staff in the OR is more used to and highly dependent on working in a team than staff in other clinical areas.

### Internal construct validity

Construct validity based on the CFA and goodness-of-fit indices (SRMR, RMSEA, and CFI) showed good model fit. If a hypothesized measurement model does not show good fit, the model needs to be re-specified and re-tested [32]. According to good model fit indices, the Swedish version of the SAQ is a valid measure of safety climate in OR departments. This finding is also an indication of the internal construct validity of the SAQ. All factors were moderately to highly correlated except for stress recognition, similar to the results of the psychometric testing of the original version of the SAQ [25].

### Methodological considerations

This study represents a first testing of the SAQ for use in Swedish ORs. The development of a valid and reliable instrument is a longitudinal and multi-step process that requires numerous positive findings across different settings. In order to establish the validity and reliability of the Swedish SAQ, additional psychometric testing is needed. The internal consistency and construct validity tests were performed on a relatively small sample compared to what is required. The relatively small sample size may be seen as a weakness in this study compared to other studies with larger sample sizes [22,23,25]. Ten respondents per item are recommended in psychometric testing in order to reach a stable co-variation among the items [32]. In the present study, this criterion would have required 300 respondents, which was aimed for but not reached because of a relatively low response rate, but has to be considered when interpreting the results. Analysis of non- respondents was not possible to perform in this study.

The response rate of the present study was 63%, which was considered acceptable. The number of missing values was fairly low. The factor working conditions had the largest proportion of missing values, which is similar to results from previous research on the SAQ [22,23]. The item “This hospital deals constructively with problem physicians” had the greatest number of missing values, which might indicate that this question was found to be difficult to answer by the respondents. The factor perception of management also had a relatively high proportion of missing values, similar to the previous Norwegian study [22]. This finding might indicate that these factors need further refinement, which should be considered in future research. The questionnaire was estimated to take 15 minutes to complete. But some respondents described that this time was not available in their workplace. One aspect is that it may even take more than 15 minutes to complete the SAQ. Several respondents also stated that the instrument contained too many items, which might have had a negative impact on the response rate. To obtain a representative sample and make comparisons between professions in the peri-operative team, including physicians in the survey would have been preferable. The data collection methods differed somewhat between the included hospitals, which is a limitation of the study, as it may pose a threat to the internal validity of the study. In one hospital, a web-based survey was used for practical reasons.

## Conclusions

The Swedish translation and psychometric testing of the SAQ (OR version) has good construct validity. However, reliability analysis suggested that some items need further refinement to establish sound internal consistency. As previous research suggests, the SAQ seems to have potential as a useful tool for evaluating safety climate. However, further psychometric testing is required with larger samples to establish the psychometric properties of the instrument for use in Sweden.

## Abbreviations

SAQ: Safety Attitudes Questionnaire; ICUMAQ: Intensive Care Unit Management Attitudes Questionnaire; FMAQ: Flight Management Attitudes Questionnaire; OR: Operating room; HSOPS: Hospital Survey on Patient Safety; AORN: Association of periOperative Registered Nurses; ISPOR: International Society for Pharmacoeconomics and Outcomes Research.

## Competing interests

The authors declare that they have no competing interests.

## Authors’ contributions

CG: Initiating project, planning project, translation and adaption of the instrument, Swedish SAQ (OR version), data collection, analysis, interpretation of data, manuscript, revision of manuscript, and final approval. FYW: Analysis, interpretation of data, manuscript, revision of manuscript, and final approval. UN: Planning project, translation and adaption of the instrument, manuscript, revision of manuscript, and final approval. AE: Initiating project, planning project, translation and adaption of the instrument, analysis, interpretation of data, manuscript, revision of manuscript, and final approval. All authors read and approved the final manuscript.

## Pre-publication history

The pre-publication history for this paper can be accessed here:

http://www.biomedcentral.com/1472-6963/13/104/prepub

## Supplementary Material

Additional file 1 SAQ factors and item descriptives. Click here for file

## References

[B1] WHO 2010 http://whqlibdoc.who.int/hq/2009/WHO_IER_PSP_2009.10_eng.pdf Visited February 6

[B2] Kohn LT Corrigan JM Donaldson MS To Err is human: building a safer health system 1999 Washington DC: National Academy Press 25077248

[B3] Patientsäkerhet: vårdskador (Patient Safety: healthcare injuries) Swedish http://www.socialstyrelsen.se/Lists/Artikelkatalog/Attachments/8780/2008-126-36_200812637.pdf

[B4] European society for quality in healthcare 2012 http://www.hope.be/03activities/docsactivities/SIMPATIE_Patient_safety_vocabulary_Professionals.pdf

[B5] Wachter R Understanding patient safety 2008 McGraw-Hill

[B6] Reason J Managing the risks of organizational accidents 1997 Surrey: Ashgate

[B7] WHO 2011 http://whqlibdoc.who.int/publications/2008/9789241596541_eng.pdf Visited February 6

[B8] Vincent C Patient safety 2010 Chichester: Wiley-Blackwell

[B9] Guldenmund FW The nature of safety culture: a review of theory and research Saf Sci 2000 34 215 257 10.1016/S0925-7535(00)00014-X

[B10] Hale A Culture’s confusions Saf Sci 2000 34 1–3 1 14

[B11] Mearns KJ Flin R Assessing the state of organizational safety – culture or climate? Current Psychology: Developmental – Learning – Personality – Social 1999 18 1 5 17 10.1007/s12144-999-1013-323543066

[B12] Nieva V Sorra J Safety culture assessment: a tool for improving patient safety in health care organizations Quality of Safety Health Care 2003 12 17 23 10.1136/qhc.12.suppl_2.ii17 PMC1765782 14645891

[B13] Robb G Seddon M Measuring the safety culture in a hospital setting: a concept whose time has come? Journal of the New Zealand Medical Association 2010 123 1313 66 76 20581914

[B14] Colla JB Bracken AC Kinney LM Weeks WB Measuring patient safety climate: a review of surveys Quality and Safety in Healthcare 2005 14 364 366 10.1136/qshc.2005.014217 PMC1744072 16195571

[B15] Pronovost PJ Goeschel CA Marsteller JA Sexton JB Pham JC Berenholz S Framework for patient safety research and improvement Circulation 2009 119 330 337 10.1161/CIRCULATIONAHA.107.729848 19153284

[B16] Vincent CA Taylor-Adams S Stanhope N Framework for analyzing risk and safety in clinical medicine Br Med J 1998 316 1154 1157 10.1136/bmj.316.7138.1154 9552960 PMC1112945

[B17] Alfredsdottir H Bjornsdottir K Nursing and patient safety in the operating room J Adv Nurs 2007 61 1 29 37 10.1111/j.1365-2648.2007.04462.x 18173734

[B18] Kingdon B Halvorsen F Perioperative nurses’ perceptions of stress in the workplace AORN J 2006 84 4 607 614 10.1016/S0001-2092(06)63939-2 17153260

[B19] Chard R How perioperative nurses define, attribute causes of, and react to intraoperative nursing errors AORN J 2010 91 1 132 145 10.1016/j.aorn.2009.06.028 20102810

[B20] Chen CK Lin C Wang SH Hou TH A study of job stress, stress coping strategies, and job satisfaction for nurses working in middle – level hospital operating rooms J Nurs Res 2009 17 3 199 210 10.1097/JNR.0b013e3181b2557b 19738448

[B21] Association of periOperative Registered Nurses (AORN) 2011 2011 http://www.aorn.org/PracticeResources/AORNPositionStatements/#axzz2AaYeI09R Visited April 5

[B22] Deilkås ET Hofoss D Psychometric properties of the Norwegian version of the safety attitudes questionnaire (SAQ), generic version (Short Form 2006) BMC Heal Serv Res 2008 8 1 10 10.1186/1472-6963-8-1 PMC2572612 18808693

[B23] Norden-Hägg A Sexton JB Kälvemark-Sporrong S Ring L Kettis-Lindblad Å Assessing safety culture in pharmacies: The psychometric validation of the safety attitudes questionnaire (SAQ) in a national sample of community pharmacies in Sweden BMC Clin Pharmacol 2010 10 8 2 12 20380741 10.1186/1472-6904-10-8 PMC2868807

[B24] Kaya S Barsbay S Karabulut E The Turkish version of the safety attitudes questionnaire: psychometric properties and baseline data Quality and Safety in Healthcare 2010 19 6 572 577 10.1136/qshc.2008.032003 20671082

[B25] Sexton JB Helmreich RL Neilands TB Rowan K Wella K Boyden J Roberts PR Thomas EJ The safety attitudes questionnaire: psychometric properties, benchmarking data, and emerging research BMC Heal Serv Res 2006 6 44 1 10 10.1186/1472-6963-6-44 PMC1481614 16584553

[B26] Sexton JB Thomas TJ Helmreich RL Error, stress and teamwork in medicine and aviation: cross sectional surveys Br Med J 2000 320 745 749 10.1136/bmj.320.7237.745 10720356 PMC27316

[B27] Helmreich RL Merritt AC Sherman PJ Gregorich SE Wiener EL The flight management attitudes questionnaire (FMAQ) NASA/ UT/FAA Technical report 1993 Austin, TX: The University of Texas 93 94

[B28] Pronovost PJ Berenholz S Goeschel C Thom I Watson S Holzmuller C Lyon JS Lubomski LH Thompson DA Needham D Hyzy R Welsh R Roth G Bander J Morlock L Sexton JB Improving patient safety in intensive care units in Michigan J Crit Care 2008 23 2 201 221 10.1016/j.jcrc.2007.09.002 18538214

[B29] Sexton JB Makary MA Tersigni AR Pryor D Hendrich A Thomas EJ Holzmuller CG Knight AP Wu Y Pronovost PJ Teamwork in the operating room: frontline perspectives among hospitals and operating room personnel Anesthesiology 2006 105 5 877 884 10.1097/00000542-200611000-00006 17065879

[B30] Modak I Sexton JB Lux TR Helmreich RL Thomas EJ Measuring safety culture in the ambulatory setting: the safety attitudes questionnaire- ambulatory version J Gen Intern Med 2007 22 1 5 17351834 10.1007/s11606-007-0114-7 PMC2227589

[B31] Wild D Grove A Martin M Eremenco S McElroy S Verjee-Lorenz A Erikson P Principles of good practice for the translation and cultural adaption process of patient-reported outcomes (PRO) measures: report of the ISPOR task force for translation and cultural adaption Value Health 2005 8 5 94 104 15804318 10.1111/j.1524-4733.2005.04054.x

[B32] Polit DF Beck CT *Nursing research. Generating and assessing evidence for nursing practice (8^th^ ed.)* 2008 Lippincot Williams & Wilkins: Crawfordsville

[B33] Rubin DB Multiple imputation for non response in surveys 1987 New York: John Wiley & Sons

[B34] Brown TA Confirmatory factor analysis for applied research 2006 New York: The Guilford Press

[B35] Bartholomew D Steele F Galbraith J Moustaki I Analysis of multivariate social science data 2008 2 Routledge: Chapman & Hall

[B36] Jöreskog K Sörbom D LISREL 8: User’s reference guide 2006 Lincolnwood: Scientific Software International

[B37] Nunnaly J Bernstein I Psychometric theory 1994 3 New York: McGraw Hill

